# Papillomavirus Vaccination Programs and Knowledge Gaps as Barriers to Implementation: A Systematic Review

**DOI:** 10.3390/vaccines13050460

**Published:** 2025-04-25

**Authors:** Giovanni Cangelosi, Francesco Sacchini, Stefano Mancin, Fabio Petrelli, Antonella Amendola, Clara Fappani, Marco Sguanci, Sara Morales Palomares, Francesco Gravante, Gabriele Caggianelli

**Affiliations:** 1 Experimental Medicine and Public Health Unit, School of Pharmacy, University of Camerino, 62032 Camerino, Italy; giovanni01.cangelosi@unicam.it; 2 Department of Nursing, Polytechnic University of Ancona, 60121 Ancona, Italy; francescosacchini@libero.it; 3 IRCCS Humanitas Research Hospital, Via Manzoni 56, Rozzano, 20089 Milan, Italy; stefano.mancin@humanitas.it; 4 Department of Health Sciences, Università degli Studi di Milano, 20146 Milan, Italy; clara.fappani@unimi.it; 5 A.O. Polyclinic San Martino Hospital, 16132 Genova, Italy; sguancim@gmail.com; 6 Department of Pharmacy, Health and Nutritional Sciences (DFSSN), University of Calabria, 87036 Rende, Italy; sara.morales@unical.it; 7 Local Health Authority of Caserta, San Giuseppe Moscati Hospital, 81031 Aversa, Italy; francesco.gravante@aslcaserta.it; 8 Azienda Ospedaliera San Giovanni Addolorata, 00184 Rome, Italy; caggianelligabriele@gmail.com

**Keywords:** human papillomavirus vaccination program, public health, health prevention, barriers, systematic review

## Abstract

Background/Objectives: Human papillomavirus (HPV) is a leading cause of cervical cancer. Despite the proven effectiveness of vaccination programs, global coverage remains uneven, with significant disparities across regions due to various socioeconomic, cultural, and political factors. This study explores the primary barriers to HPV vaccination worldwide and proposes recommendations to improve access to screening and vaccination programs. Methods: A systematic literature review was conducted, analyzing studies published in the past ten years from databases such as PubMed, Scopus, and Embase, following the PRISMA methodology. Study selection involved multiple researchers, with discrepancies resolved through consultation. The quality of the included studies was assessed using CASP checklists. The protocol was registered on Open Science Framework (OSF). Results: Out of 2119 records, eight studies were included. The findings indicate that the main barriers to HPV vaccination include insufficient public awareness, cultural and religious resistance, financial constraints, and limited access in rural and underserved areas. Additionally, political factors, such as low prioritization of HPV vaccination and the absence of supportive policies, were identified as significant obstacles. Multidisciplinary and cross-cultural collaboration, along with the integration of HPV vaccination into existing health programs, was suggested as a strategic approach to improve vaccine uptake. Conclusions: Barriers to HPV vaccination, including limited awareness, inadequate healthcare infrastructure, and socioeconomic factors, vary across regions but must be addressed to improve global coverage. Targeted interventions such as health education, inclusive policies, and culturally sensitive campaigns can significantly boost vaccine uptake. Strengthening local health systems and fostering international collaboration are key strategies to overcoming these barriers and ensuring equitable access to HPV vaccination.

## 1. Introduction

Human papillomavirus (HPV) is one of the most common sexually transmitted infections worldwide, with a high prevalence among sexually active individuals [1]. HPV is associated with several cancers, particularly cervical cancer, as well as anal, vulvar, and oropharyngeal cancers [1,2]. The World Health Organization (WHO) has identified over 200 HPV types, with high-risk strains being responsible for cancer in some cases [3]. Although most HPV infections are asymptomatic and resolve spontaneously, persistent infections with high-risk types can lead to malignancies [4,5]. WHO estimates that approximately 662,044 new cervical cancer cases were diagnosed in 2022, resulting in around 350,000 deaths globally [6]. The 5-year relative survival rate for cervical cancer varies depending on the stage at diagnosis [7]. For cases diagnosed at a localized stage, the 5-year survival rate is 91.1%, decreasing to 60.8% for cases with regional lymph node involvement and dropping to 19.4% for cases with distant metastasis [2,8,9].

The primary treatment for HPV-related diseases, particularly cervical cancer, involves a combination of surgery, radiation therapy, and chemotherapy, depending on the cancer stage [10]. However, prevention remains the most effective strategy. Currently, six HPV vaccines have been licensed, differing according to vaccine composition (number of genotypes they protect against). All vaccines are protective against high-risk HPV types 16 and 18, associated with approximately 70% of cervical cancer cases globally, and are highly effective in preventing HPV infection and, consequently, reducing the incidence of HPV-related cancers, including cervical, anal, and oropharyngeal cancers. These vaccines are licensed for use in both females and males starting from 9 years of age, depending on national immunization policies and regulatory approvals. According to WHO recommendations, girls aged 9–14 years who are not yet sexually active represent the primary target population for HPV vaccination. The secondary target population consists of females aged ≥15 years and males. Vaccination of this population is recommended only if it is feasible and affordable and does not divert resources from vaccination of the primary target population [11].

In addition to vaccination, regular screening programs, such as Pap smears and HPV testing, are vital for the early detection of precancerous cervical lesions [12]. International data strongly support the effectiveness of screening programs in reducing cervical cancer incidence and mortality [10,13]. For instance, countries with well-organized screening programs have observed a significant reduction in cervical cancer rates [14]. The combination of screening and vaccination provides a dual approach to prevention and early intervention, significantly improving clinical outcomes [10,15].

Vaccination safety has been extensively studied, with no significant long-term adverse effects identified in large-scale trials [16]. The benefits of vaccination far outweigh its minimal risks, offering strong protection against high-risk HPV infections and significantly reducing the burden of HPV-related diseases [17]. By incorporating vaccination and regular screening into public health strategies, the global incidence of HPV-related cancers can be substantially reduced, leading to better health outcomes and improved quality of life [10,18,19].

Despite the proven effectiveness of HPV vaccination and screening programs, global resistance remains a significant barrier to widespread adoption [13]. Resistance to these programs varies between regions and is influenced by cultural beliefs, political views, and socioeconomic challenges [20]. In some areas, misinformation about vaccine safety and distrust in health authorities have contributed to lower vaccination rates [10,21]. For example, in certain African and Southeast Asian countries, HPV vaccination uptake remains relatively low [22]. The global North and South disparities in vaccine dose viability represent another key issue in reaching optimal vaccine coverage. The disparity is associated with both logistical barriers (e. g., maintaining the cold chain between production factories and healthcare facilities) and economic constraints; indeed, HPV vaccines are among the most expensive vaccines currently available [23,24].

Moreover, screening programs face challenges, particularly in low-resource settings, where limited healthcare access, lack of awareness, and insufficient infrastructure hinder participation [25]. A systematic review of global literature highlights inconsistencies in data regarding public resistance to vaccination and screening, with varying levels of acceptance reported across different studies. Factors such as fear of side effects, religious or cultural opposition, and logistical barriers (e.g., cost, limited availability of healthcare providers) significantly impact adherence to these programs [26].

This variability in resistance is further compounded by a lack of standardized data across regions, making it challenging to develop tailored interventions. Addressing these barriers requires a multifaceted approach, incorporating education, policy support, and improved healthcare access to promote the widespread adoption of vaccination and screening programs globally [10,16].

This study contributes significantly to the scientific community by addressing gaps in the literature related to HPV vaccination programs and the barriers to vaccine uptake. While substantial evidence supports the efficacy of HPV vaccines [27,28], limited research specifically focuses on identifying and analyzing the barriers to vaccination. To date, no recent systematic reviews have comprehensively explored this topic.

Most existing studies concentrate on the clinical effectiveness of HPV vaccines without providing in-depth insights into the challenges hindering global vaccination coverage [10].

The primary study aimed to identify and analyze global barriers to HPV vaccination, with a focus on the knowledge gap and reporting examples from the different countries, populations, and healthcare workers involved in the awareness-raising process and management of the clinical pathway. Secondarily, the study aims to analyze these barriers and knowledge gaps in order to suggest possible future research strategies towards the population, healthcare workers, and health systems involved in the management and containment of HPV infection.

## 2. Materials and Methods

### 2.1. Design and Registration

A systematic review of the literature on HPV vaccination programs and the barriers to their implementation was conducted to ensure methodological rigor and the relevance of the selected studies. This review followed the Preferred Reporting Items for Systematic Reviews and Meta-Analyses (PRISMA) guidelines (Supplementary File S1: PRISMA Checklist) [29,30]. The protocol for this review was registered in the Open Science Framework (OSF) database (https://doi.org/10.17605/OSF.IO/SWVKF).

### 2.2. Eligibility Criteria

The inclusion criteria encompassed primary studies published in English within the last 10 years, relevant to the study’s objectives, and involving individuals over 18 years old in community-based settings. Exclusion criteria eliminated other types of studies (e.g., editorials, commentaries, reviews, and protocol studies). Studies involving school-based contexts or parents, as well as studies written in Chinese, were also excluded.

### 2.3. Information Sources

A comprehensive and systematic search for relevant literature from the past ten years has been conducted across three databases: PubMed/Medline, Scopus, and Embase. This methodical approach aimed to capture a broad and diverse range of perspectives and sources in the field on HPV vaccination programs and associated barriers.

### 2.4. Search Strategy and Research Questions

The search strategy was developed using the PICOS framework (Population, Intervention, Comparison, Outcome, Study Design) [31], and was defined as follows: P, adults > 18 years; I, HPV vaccination programs in community settings; C, absence of adequate programs or presence of barriers; O, increased vaccination coverage and reduced barriers to access; and S, primary studies. The search strategy involved the use of keywords such as “Healthcare Disparities”, “Mass Vaccination”, and “Papillomavirus Vaccines” with a combination of Boolean operations such as AND/OR (Supplementary File S2: Search strategy).

The search strings were adapted to meet the specific requirements of each database, ensuring efficient literature retrieval. These keywords were designed to target the relevant search scope in line with our eligibility criteria. Following the methodological standards [29,30], we have provided a complete account of our search strategy, including the final search strings for each database.

The review aimed to address the following research questions:

Primary:

What are the global barriers to HPV vaccination, particularly in relation to knowledge gaps and country-specific differences?

Secondary:

What are the global barriers to HPV vaccination, particularly regarding knowledge gaps and country-specific differences, and how can their analysis inform future research strategies?

What are the differences and affinities in HPV program implementation between high-income and low- and middle-income countries, with particular attention to population and healthcare professionals’ knowledge, as well as resource allocation and management?

### 2.5. Selection Process

The study selection process for this review followed a two-phase procedure: an initial screening of titles and abstracts, followed by a detailed evaluation of full-text articles. All potentially relevant articles were imported into the reference manager Mendeley (free version 2.120.0) for organization and management [32]. Both automated and manual techniques within Mendeley were utilized to remove duplicates, ensuring the thorough elimination of redundant records. The initial screening was conducted independently by two authors [G.C. (Giovanni Cangelosi) and F.S.], who evaluated titles and abstracts based on their relevance to the study and in accordance with the predefined inclusion criteria: original research articles published in peer-reviewed journals, written in English, and focused on the PICOS framework. Studies were excluded if they were reviews, editorials, or conference abstracts, were not in English, or did not directly addressing the research question. A third independent researcher [G.C. (Gabriele Caggianelli)] resolved any disagreements at this stage. Following the initial screening, full-text articles meeting the preliminary criteria were independently assessed by [G.C. (Giovanni Cangelosi) and F.S.] according to the predefined eligibility standards. Any discrepancies were resolved through consensus meetings, with [G.C. (Gabriele Caggianelli)] acting as an arbitrator to ensure integrity in the selection process. This systematic approach ensured a rigorous and unbiased selection of studies for the review.

### 2.6. Data Collection Process

The data extraction process was carefully designed to ensure the accurate and reliable information collection from the selected studies. Mendeley software (free version, Free V 2.132.1) was used to manage references and facilitate data extraction [32]. Two independent reviewers [G.C. (Giovanni Cangelosi) and F.S.] extracted data using a standardized data collection form developed a priori. This dual-reviewer approach (in blind), as recommended in methodological guidelines [29,30], was adopted to minimize bias and improve data reliability. Articles with missing or unclear data were noted, and where necessary, authors were contacted for clarification. In cases of discrepancies or differing interpretations, a third researcher [G.C. (Gabriele Caggianelli)] was consulted to resolve the issues, ensuring the integrity of the data extraction process.

### 2.7. Data Items

Data extraction from the included studies was organized into key categories, consistent with the methodological framework [29,30]. This structured categorization facilitated both detailed reporting and thorough analysis. The main categories included bibliographic details (authors and publication year), study design, sample characteristics, primary intervention, limitations, and key findings. This structured approach enhanced the clarity and depth of our analysis, aligning with the established methodological standards. The extracted data were presented as a narrative summary, organized according to the review’s objectives and supplemented with Figures and Tables.

### 2.8. Study Risk of Bias Assessment

The risk of bias and the methodological quality of the included studies were assessed using established guidelines [33]. Two independent reviewers [G.C. (Giovanni Cangelosi) and F.S.] conducted the evaluation to ensure objectivity. Any disagreements were resolved through discussions with a third author [G.C. (Gabriele Caggianelli)], ensuring that a consensus was reached. The risk of bias and the methodological quality of the included studies were evaluated using the Critical Appraisal Skills Programme (CASP) checklists (Supplementary File S3) [33]. The CASP checklist assesses the clarity and robustness of the study design, including sample selection, data collection methods, and tool reliability. It also evaluates the transparency and replicability of the methods to ensure reliable and reproducible results.

### 2.9. Effect Measures

The synthesis and presentation of the study results followed established guidelines [29,30]. This process involved extracting and analyzing quantitative data from the selected studies to ensure a comprehensive and precise representation of the findings. Key statistical measures, including means (M), standard deviations (SD), and *p*-values, were integral to the analysis. To maintain the integrity of the original studies, statistical significance reporting was preserved as presented in each study. Consistent with scientific conventions, *p*-values of 0.05 or lower were considered statistically significant, ensuring the inclusion of robust and meaningful findings in the review.

### 2.10. Synthesis of Results

In this review, while the benefits of meta-analysis are acknowledged, a combined quantitative synthesis was deemed not feasible due to the heterogeneity of the included studies. This variability, characterized by differences in intervention types and methodologies for quantifying relationships between variables, led to inconsistencies in both the methodological and statistical approaches. As a result, a detailed narrative synthesis was chosen, following established guidelines for synthesis without meta-analysis (SWiM) [34]. This approach was selected for its effectiveness in transparently and rigorously synthesizing diverse quantitative data, aligning with PRISMA guidelines [29,30].

## 3. Results

**Figure 1:**
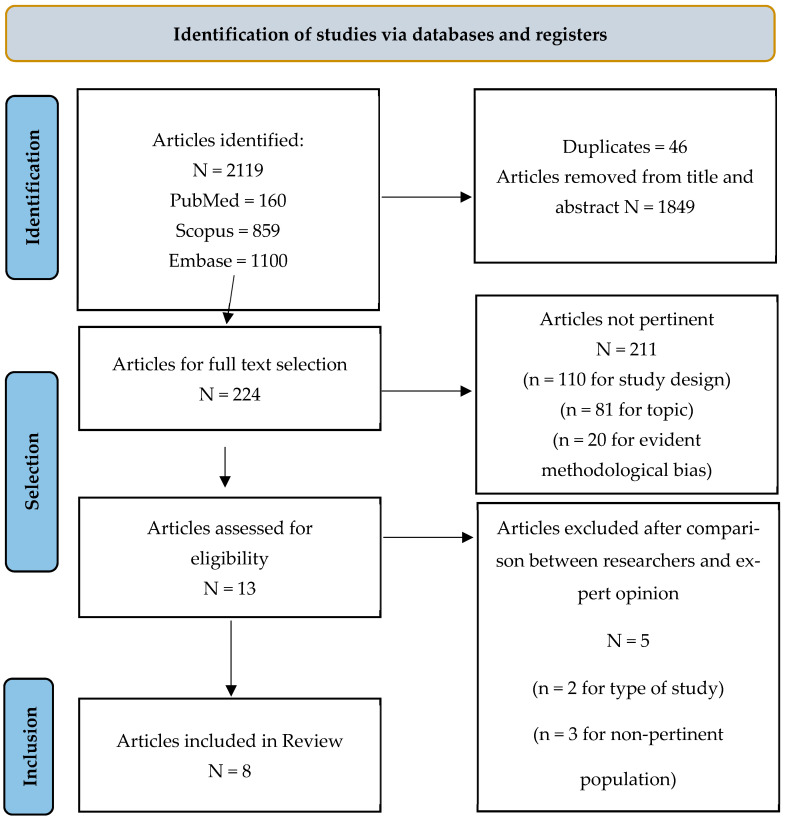
PRISMA Flow Chart.

The search strategy identified 2119 articles from the PubMed-Medline, Embase, and Scopus databases. After the removal of 46 duplicates and the exclusion of 1849 articles based on title and abstract screening, 224 articles were selected for full-text assessment. Among these, 211 articles were deemed irrelevant (110 for study design, 81 for topic, and 20 for evident methodological bias), and 13 full-texts were assessed for eligibility. Following further review and expert opinion, five additional articles were excluded (Supplementary File S1). The final selection process resulted in the inclusion of eight primary studies, conducted between 2015 and 2024. The PRISMA flowchart is presented in Figure 1, and a summary of the included studies is provided in Table 1.

### 3.1. Characteristics of the Included Studies

Among the eight primary studies [35,36,37,38,39,40,41,42], five were conducted in North America [36,37,38,39,40], two in Africa [35,41], and one in Oceania [42]. Of these, seven were observational studies [35,36,37,39,40,41,42], while one was a randomized controlled trial (RCT) [38]. Four studies focused on HPV vaccination education and awareness [35,36,39,41], two investigated attitudes and perceived barriers to HPV vaccination [37,40], and two assessed interventions and factors influencing HPV vaccination uptake [38,42].

### 3.2. Systematic Results of the Included Studies

#### 3.2.1. HPV Vaccination Awareness and Education

A cross-sectional study [35] conducted in 23 African countries examined 153 healthcare providers involved in cervical cancer prevention. Participants completed an online survey assessing their education, knowledge, and propensity to recommend the HPV vaccine. Findings revealed that only 37.4% had the vaccine available in their facility, while 56.2% had not received specific training on its administration. Despite this, 83.2% of participants recommended the vaccine, primarily for girls (82.1%). The main reasons for not recommending it included its unavailability (57.1%) and a lack of effective communication tools (28.6%). Additionally, only 9.9% of providers correctly answered all vaccine-related knowledge questions, highlighting a general lack of knowledge. In contrast, an observational study [36] conducted in the United States involved 223 primary care physicians, who were invited to participate in an online educational intervention aimed at improving HPV vaccination practices. Of these, 65% (144 participants) completed the module, which was evaluated through pre-test, post-test, and delayed post-test assessments. Results indicated a significant increase in knowledge scores from pre-test to post-test (mean 3.8 to 4.6 out of 5), with a notable improvement among internists (3.6 to 4.6). In addition, physicians reported increased confidence in recognizing patients needing vaccination in the 19–26 and 27–45 age groups, with increased confidence scores (from 3.3 to 3.7 and 2.7 to 3.5, respectively). There was also a significant increase in the frequency of counseling on the risks of HPV infection, with an increase in the mean score (from 2.3 to 2.8). Improvements in knowledge and confidence were sustained over time, as evidenced by the delayed post-test assessment. Participants highly recommended the course, with a mean rating of 4.5 out of 5. In parallel, in a qualitative study [41] conducted in a rural hospital in Zimbabwe, a court of 15 healthcare providers was surveyed to assess the acceptability and uptake of HPV vaccines. Participants, including nurses, physicians, and counselors, were surveyed to understand their perceptions of current hospital practices in cervical cancer prevention and treatment, their knowledge of HPV and HPV vaccines, and their views on the introduction of HPV vaccination programs. Despite a general lack of knowledge about HPV and HPV vaccines, strong support emerged for the implementation of vaccination programs. Healthcare providers identified numerous psychosocial, cultural, and logistical barriers to effective implementation and proposed several culturally appropriate solutions, such as education and community engagement. Healthcare providers were supportive of HPV vaccination in Zimbabwe, but still identified psychosocial, cultural, and logistical barriers such as cost, schedule, and healthcare infrastructure. Finally, a multicenter qualitative observational study [39] conducted in the United States examined how collaborations between clinical and community entities can promote HPV vaccination and improve vaccination rates through community–clinic linkages (CCLs). Interventions focused on community engagement to raise awareness about HPV vaccination, with administration and follow-up managed primarily in clinical settings. The main barriers included a lack of HPV awareness, technology communication problems between partners, and administrative challenges. Results demonstrated that CCLs effectively improve vaccination coverage, suggesting that integrating community services with clinical settings can bridge gaps in coverage through a coordinated health promotion and intervention strategy. Strategic, well-organized partnerships between communities and clinics were deemed critical to maintaining high levels of HPV vaccine coverage. HPV vaccination lacks institutional prioritization and suffers from documentation issues, limiting accurate tracking and adequate resource allocation.

#### 3.2.2. Attitudes and Barriers Toward HPV Vaccination

A qualitative observational study [37] conducted in the United States involved 16 semi-structured interviews with patients using pre-exposure prophylaxis (PrEP). The study explored attitudes, beliefs, and perceived barriers to HPV vaccination. Participants generally viewed vaccination as an important preventive measure and were supportive of receiving it, provided it was recommended by their physician and covered by insurance. However, significant gaps in knowledge emerged regarding the effects of HPV, particularly its impact on men. Many participants believed that HPV posed a greater risk for women. Major barriers to vaccine acceptance included lack of awareness of guidelines regarding the appropriate age for vaccination, lack of explicit medical recommendations, and insufficient insurance coverage. Despite these challenges, vaccine acceptance was high: 100% of eligible unvaccinated participants agreed to be contacted for vaccination arrangements. In parallel, another observational study [40] in the United States analyzed a cohort of 1457 unvaccinated women using data from the 2010 National Health Interview Survey to investigate the impact of relationship status on interest in HPV vaccination. Among the participants, only 31.4% expressed interest in receiving the vaccine. The analysis indicated that cohabiting women had a 45% higher probability of being interested in vaccination than married women (PR = 1.45, 95% CI 1.06–1.90), while single women exhibited a 42% increase in the probability of interest compared to married women (PR = 1.42, 95% CI 1.11–1.76). In addition, the main reasons for not vaccinating differed significantly by relationship status: 47.6% of married women cited lack of need for the vaccine as the main reason, compared to 34.7% of never-married women. Concerns about vaccine safety were less frequent among married women (3.9%) than among those living with a partner (15.2%) and those never married (14.9%).

#### 3.2.3. Interventions and Success Factors in Vaccine Adoption

A RCT [38], was conducted across eight clinics in the United States, enrolling 217 women, who were randomized into two groups: the experimental group (EG) (n = 136) and the control group (CG) (n = 81). The EG received an intervention called “Women’s Stories” (WS), a program based on personalized narratives designed to promote HPV vaccination, delivered through touch-screen tablets in waiting rooms. The primary outcomes assessed were immediate, 1-month, and 6-month vaccination intentions, as well as vaccination self-efficacy. Results demonstrated a significant increase in vaccination intentions and self-efficacy in the EG compared with the CG, with *p* < 0.01 for immediate, 1-month, and 6-month vaccination intentions, and B = 0.54; *p* = 0.0002 for self-efficacy. Additionally, 41% of participants in the EG reported being extremely confident in completing the three-dose HPV vaccination series, compared to 29% in the CG. In an observational study [42] conducted in Australia, 1139 women were analyzed to assess factors associated with HPV vaccine adherence. Participants completed a self-administered questionnaire regarding their vaccination status, sociodemographic characteristics, and behavioral factors. Findings indicated that 77% (n = 880) received at least one dose of the vaccine, while 68% (n = 777) received at least two doses. The analysis showed that women born in Australia were significantly more likely to be vaccinated than those born abroad (*p* < 0.01). Being single (*p* = 0.02), nulliparous (*p* < 0.01), and living in areas with higher socioeconomic status (*p*-trend = 0.03) or in more remote areas (*p* = 0.03) were also factors associated with higher vaccination rates. Behavioral factors such as alcohol consumption (*p* < 0.01) and hormonal contraceptive use (*p* < 0.01) were associated with a higher likelihood of vaccination. Although vaccinated women tended to have fewer sexual partners than unvaccinated women (*p*-trend = 0.02), they were also more likely to report a previous sexually transmitted infection (STI) (*p* = 0.03).

## 4. Discussion

HPV vaccination plays a fundamental role in reducing the incidence of cervical carcinoma and other HPV-related diseases [43]. However, global vaccine uptake remains suboptimal, with significant regional disparities [44]. Identifying and analyzing the barriers to vaccine coverage is essential for developing effective strategies that improve accessibility and adherence to vaccination, and consequently enhance the overall effectiveness of HPV vaccination programs. This is essential to be in line with the WHO goal to eliminate cervical cancer as a public health problem by the end of the twenty-first century, which requires vaccinating 90% of girls all over the world with the HPV vaccine by the age of 15 years by 2030 [23].

All over the world, the implementation of effective vaccination programs requires overcoming structural, social, and organizational challenges. Adequate and stable funding is essential to ensure the sustainability of the vaccine program and the quality of services delivered. However, the availability of economic resources is uneven across countries, directly affecting their capacity to build new immunization programs and to maintain and expand existing ones. At the same time, regulatory and cultural barriers restrict access to vaccines for vulnerable populations such as refugees, migrants, and women—particularly in contexts where unfavorable gender norms or discriminatory practices hinder their autonomy in health-related decisions [45].

One of the primary barriers to HPV vaccination is limited awareness and understanding of its importance within communities, particularly in low- and middle-income countries (LMICs) [35,46,47]. A systematic review conducted by Ver et al. [48] highlighted knowledge gaps regarding HPV and its vaccine among healthcare professionals and potential recipients in the Asia–Pacific region.

Similar findings were reported in Latin America, where Nogueira-Rodrigues et al. [49] found that misconceptions about HPV transmission and vaccine safety have hindered vaccination efforts. These knowledge gaps often reflect systemic issues, including limited access to medical education and lack of institutional communication strategies. Other than lack of education, these knowledge gaps may result from limited access to reliable health communication channels, especially in under-resourced settings. In this context, community-based interventions emerged as key strategies in overcoming such barriers [39,41]. For example, the success of community–clinic linkages in the United States [39] and community engagement strategies proposed in Zimbabwe [41] point to scalable models that integrate culturally sensitive communication, trust-building, and logistical support.

These knowledge deficits are further exacerbated by cultural stigma surrounding STIs, influencing parental willingness to vaccinate their children [19,37,38]. Although the studies included in our review did not report this aspect in detail, the systematic reviews by MacDonald et al. and Islam et al. [50,51] highlight how societal taboos around sexuality and sexually transmitted infections can discourage open dialogue and reinforce vaccine hesitancy. In many cultures, discussing sexual health with adolescents is still considered inappropriate, creating a psychological barrier to acceptance of a vaccine perceived to be linked with sexual activity. These insights provide useful context to understand the indirect mechanisms influencing vaccination behavior.

In many regions, particularly sub-Saharan Africa, weak healthcare infrastructure poses significant challenges to HPV vaccination programs [36,41]. Studies conducted by Amchas-Dacosta et al. [52] and Kutz et al. [53] provide substantial evidence that inadequate healthcare systems, a lack of trained personnel, and disruptions in vaccine supply chains severely limit vaccination coverage. Waheed et al. [54] further emphasize that these structural issues often coincide with systemic barriers, such as underfunding and policy deficiencies that fail to prioritize HPV vaccination. A similar situation is observed in the Eastern Mediterranean region, where Hakimi et al. [55] documented comparable systemic healthcare delivery barriers affecting vaccine uptake. The interface between the logistics and the policy has a key role in building sustainable vaccine delivery programs. Vaccine cold chain maintenance, for instance, requires both infrastructure and trained personnel—resources often unavailable in remote or economically disadvantaged regions.

Socioeconomic factors also play a crucial role in shaping access to vaccination [42]. According to Xu et al. [56], economic constraints, including the high cost of vaccination and transportation barriers, disproportionately affect marginalized communities, limiting their access to healthcare services. Additionally, geographical disparities present further challenges; rural populations often experience greater accessibility issues than urban residents [57]. In the United States, for instance, rural communities report lower HPV vaccination rates, partly due to these inequalities [58]. Rural residents may need to travel long distances to reach vaccination centers, which not only increases direct costs (e.g., transport, time off work) but also indirectly reduces motivation. Moreover, individuals in such areas may have fewer interactions with health professionals who could recommend the vaccine.

Cultural beliefs and practices further complicate vaccination efforts with HPV [39,40]. Specific attitudes of the community towards vaccination can create an environment in which the absorption of the vaccine is perceived negatively. It has been discovered that among Indigenous populations all over the world, the variations in cultural beliefs regarding sexual health have significantly influenced their attitudes against the HPV vaccine, as identified in a systematic review of MacDonald et al. and Islam et al. [50,51]. These findings underline the importance of involving community leaders in developing culturally sensitive campaigns tailored to the local contexts in order to relieve misconceptions and encourage vaccine uptake.

The successful implementation of HPV vaccination programs often requires region-specific strategies. Branda et al. [59] advocate for a multifaceted approach that includes educational campaigns to increase awareness among healthcare workers and communities. Advanced training programs for healthcare professionals are essential to ensure they can effectively address public concerns regarding vaccine safety and efficacy [60]. Such interventions are particularly relevant in regions where trust in the healthcare system is low and the healthcare system is perceived as externally imposed, as demonstrated by experiences in sub-Saharan Africa [52]. While educational initiatives are crucial, logistical improvements are equally important. Tsu et al. [61] support policy innovations that streamline vaccine distribution and improve the logistical capabilities of healthcare systems in LMICs. Such improvements may include partnerships with local organizations and leveraging existing healthcare infrastructure to facilitate mobile vaccination units in hard-to-reach areas. Additionally, significant investments in public health infrastructure are required to support sustainable vaccination efforts and enhance program resilience against disruptions [62]. The role of government policies cannot be overstated. As highlighted by Wang et al. [63], eliminating financial and regulatory barriers is essential for creating a supportive environment for HPV vaccination. Developing national immunization policies that prioritize HPV vaccination can help overcome financial constraints and reduce inequities in vaccine access. Governments must collaborate closely with international organizations to secure funding and technical support for successful implementation [64]. Furthermore, understanding the social determinants of health that influence vaccine uptake is key to designing community-specific interventions. A narrative review by Essa-Hadad et al. [65] found that minority and marginalized populations often face unique barriers to vaccination, necessitating targeted interventions that address their specific needs. Strategies such as mobile clinics, culturally sensitive awareness campaigns, and educational programs can help bridge the gap in vaccine coverage. Additionally, improving vaccination coverage requires evidence-based quality improvement programs [66]. A comprehensive understanding of the barriers to HPV vaccination, particularly regional disparities, is crucial for improving global vaccine coverage. The evidence collected in this review highlights how provider education, community engagement, and integrated delivery models are promising pathways for intervention. Addressing knowledge gaps, strengthening healthcare infrastructure, and enhancing logistical capabilities can make vaccination programs more accessible and effective. Research and policy innovations should be aligned with community needs and cultural contexts to build trust and increase participation in HPV vaccination efforts. These strategies are essential to reducing the burden of HPV-related diseases and achieving significant progress in global public health.

### 4.1. Perspectives for Future Research, Clinical Practice, and Policy Raccomandations

**Figure 2:**
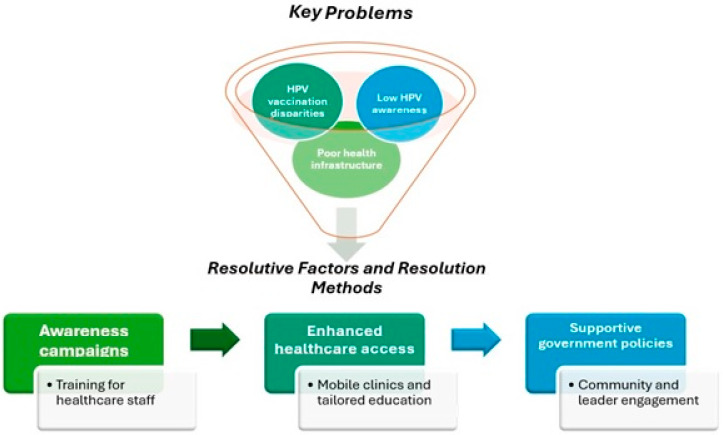
Perspectives for Future Research, Clinical Practice, and Policy Recommendations.

The findings of this study, summarized in Figure 2, have significant scientific and professional implications. Scientifically, they highlight the complex, multifactorial barriers to HPV vaccination, emphasizing the need for further research on tailored interventions addressing sociocultural, economic, and political factors. However, while the study identifies well-known barrier factors, it falls short in detailing how these findings can be translated into actionable strategies or policy initiatives, which may limit its immediate practical applicability. A recent study estimated that an increase by 56.8% and 80.7% for cervical cancer cases and deaths, respectively, from 2022 to 2050 will be necessary to increase attention on this issue globally [14]. Professionally, the study underscores the importance of integrating HPV vaccination into broader health systems and policy frameworks, suggesting that healthcare professionals should advocate for inclusive, culturally sensitive strategies. In this regard, considering the substantial heterogeneity across regions in terms of political priorities, economic resources, and social attitudes toward vaccination, the identification of context-specific barriers could inform more nuanced and regionally adapted policy recommendations [67,68,69]. Additionally, this research encourages collaboration between healthcare providers and policymakers to strengthen vaccination programs and improve access to cervical cancer prevention, particularly in underserved regions, to reduce access disparities [70,71]. Finally, as has long been established in the management of chronic conditions both in clinical, nursing, and social contexts [72,73,74,75,76,77], the potential development of advanced technologies for the management of extensive vaccination prevention programs could help reduce existing barriers to care access and improve healthcare management both in epidemiological and therapeutic terms [78,79,80]. Based on the findings of this study and the identified barriers to HPV vaccination, the following policy recommendations are proposed to improve vaccine uptake and reduce disparities in access [3,11,22,23]:

- Prioritize comprehensive public health campaigns to increase awareness of HPV and the vaccine’s importance, particularly targeting marginalized communities and underserved regions;
- Invest in healthcare infrastructure and resources to ensure the effective implementation of HPV vaccination programs, focusing on areas with limited resources;
- Implement policies that address the socioeconomic barriers to vaccine access, including financial support and improved transportation networks for vulnerable populations;
- Foster international collaboration to share best practices and develop region-specific strategies that account for local cultural, political, and economic contexts;
- Advocate for the integration of HPV vaccination into broader health policies that focus on cancer prevention, public health equity, and the improvement of healthcare delivery systems.

### 4.2. Strengths and Limitations

Our study achieved a high methodological quality according to the CASP framework, although significant limitations were identified in the selected studies [35,36,37,38,39,40,41,42], among which the following are the most relevant: sampling bias due to non-representative samples and self-reported data, selection bias, and methodological limitations, and limited generalizability of the collected data which cannot be extended to the general population. Strengths of this study include its systematic approach, which followed PRISMA guidelines and included a comprehensive review of recent literature from multiple databases. The involvement of multiple researchers in the study selection and the use of CASP checklists to assess study quality ensured a rigorous and unbiased analysis. However, a key limitation is the relatively small number of included studies (n = 8) covering a broad range of geographical regions, potentially limiting the generalizability of the findings. Moreover, it is important to consider that the studies included were conducted in different periods, and the changes in social, economic, and healthcare conditions over time may affect the applicability of these results to current or future policy frameworks. Additionally, relying solely on published studies may introduce publication bias, and the study does not provide specific quantitative data to strengthen its conclusions. Another relevant limitation that prevented a quantitative synthesis of the results through meta-analysis is the extreme heterogeneity of the included studies, both in terms of methodological design and the healthcare settings in which they were conducted. Furthermore, challenges related to vaccine availability may have led to underestimated efficacy rates, particularly in studies assessing immunization responses. Finally, the impact of the COVID-19 pandemic may have influenced participant behaviors and perceptions, introducing significant confounding variables that were often overlooked. Collectively, these methodological limitations highlight the need for more robust study designs and diverse population samples to advance knowledge in this field.

## 5. Conclusions

This study identified several critical barriers to HPV vaccination, including limited awareness, inadequate health infrastructure, socioeconomic disparities, and systemic issues that hinder access and coverage. Public knowledge about HPV and its vaccine remains limited, leading to underutilization, despite the vaccine’s proven efficacy in preventing cervical cancer and other HPV-related diseases. Inadequate health infrastructure further exacerbates this situation, as many healthcare facilities lack the necessary resources to effectively promote, administer, and manage HPV vaccination programs. Socioeconomic factors also play a significant role, disproportionately affecting marginalized populations, as financial constraints and transportation barriers limit access to vaccination services. Additionally, systemic challenges, such as lack of political support and fragmented healthcare systems, further complicate vaccine distribution and obstruct efforts to improve vaccination rates. However, these findings should be interpreted with caution, given the relatively small number of studies included in this review and their uneven geographical distribution, which may limit the generalizability and reliability of the conclusions. Moreover, the reliance on published data may introduce publication bias, and the absence of specific data supporting some conclusions could affect their overall credibility and verifiability. Another relevant aspect to consider is that the time periods in which the included studies were conducted may reflect different social, economic, and healthcare conditions, which could influence the relevance of the findings to contemporary or future policy frameworks.

These obstacles highlight the urgent need for comprehensive and integrated strategies that address structural barriers, ensuring equitable vaccine access and potentially reducing the global burden of HPV-related diseases. Overcoming these multifaceted challenges is essential to improving public health outcomes, increasing HPV vaccine uptake, and ultimately enhancing global cancer prevention efforts.

## Figures and Tables

**Table 1:** Synthesis of Included Studies.

First Author/Year/Location	Type of Study	Population	Main Intervention	Main Limitations	Main Results
Fokom Domgue et al. [35], 2024, Africa	Observational, cross-sectional	153 healthcare workers	Online survey on training, knowledge, and willingness to recommend the HPV vaccine	Representativeness bias: The study is limited to urban settings; there is limited accessibility to the vaccine.	Only 37.4% had the vaccine available in their facility; 56.2% had not received specific training; 83.2% recommended the vaccine, mainly for girls; and lack of training (28.6%) was a key reason for not recommending the HPV vaccine.
Zhang et al. [36], 2023, America	Observational	223 primary care physicians	Online educational course to improve HPV vaccination practices	Data incompleteness: lack of demographic data for early participants; low response rate for the post-intervention test.	Significant increase in knowledge and confidence scores in HPV vaccination, maintained over time.
Sullivan-Blum et al. [37], 2022, America	Observational, qualitative	16 PrEP patients	Semi-structured interviews to explore attitudes, beliefs, and perceived barriers	Small sample: only 16 interviews; lack of formal recording of interviews.	High vaccine acceptance if recommended by the physician and covered by insurance, with significant gaps in knowledge about the effects of HPV on men.
Hecht et al. [38], 2022, America	RCT	EG (n = 136) CG (n = 81)	Program based on personalized narratives to promote HPV vaccination	Lack of data on actual vaccination; limited generalizability due to the specific study population with Planned Parenthood clients.	Significant increase in vaccination intentions and self-efficacy, with 41% in the EG extremely confident of completing the vaccination cycle.
Brandt et al. [39], 2019, America	Observational, qualitative, multicenter	Clinical and community entities	Examination of community–clinic collaborations to promote HPV vaccination	Selection bias: study limited to known collaborations; methodological limitations in qualitative data collection techniques.	Improvement in vaccination rates through community–clinic coordinated interventions. Main barriers included poor knowledge of HPV and administrative challenges. HPV vaccination underfunded or poorly documented in medical records.
Thomson et al. [40], 2016, America	Observational	1457 young unvaccinated women	Data analysis from the National Health Interview Survey 2010	Recall bias due to the use of self-reported data; high non-response rate.	Only 31.4% of women expressed interest in the vaccine. The main reasons for non-vaccination varied significantly based on relationship status.
Crann et al. [41], 2016, Africa	Observational, qualitative	15 healthcare providers	Interviews to assess knowledge and opinions on HPV and HPV vaccines	Limited initial knowledge of HPV and vaccines; non-representative sample due to convenience sampling.	Strong support for the implementation of vaccination programs despite identified barriers. Supportive, but barriers included cost, schedule, and healthcare infrastructure.
Canfell et al. [42], 2015, Oceania	Observational	1139 young adult women	Self-administered questionnaire on vaccination status, sociodemographic information, and behavioral characteristics	Data reliability is compromised by self-reporting; potential overestimation of vaccine uptake due to opportunistic vaccination.	A total of 77% received at least one dose of the vaccine. Factors such as being born in Australia, being single, and living in areas with high socioeconomic status were associated with a higher vaccination rate.

Legend. HPV: Human Papillomavirus; PrEP: Pre-Exposure Prophylaxis; RCT: Randomized Controlled Trial; EG: Experimental Group; CG: Control Group.

## Data Availability

The data supporting this research are available upon request from the corresponding author for data protection reasons.

## Supplementary Materials

The following supporting information can be downloaded at: https://www.mdpi.com/article/10.3390/vaccines13050460/s1, File S1. Prisma Check List; File S2. Search strategy; File S3. Critical Appraisal Skills Programme (CASP) checklists.

## Protocol Registration

https://doi.org/10.17605/OSF.IO/SWVKF.

## References

[1] Wolf J., Kist L.F., Pereira S.B., Quessada M.A., Petek H., Pille A. (2024). Human Papillomavirus Infection: Epidemiology, Biology, Host Interactions, Cancer Development, Prevention, and Therapeutics. Rev. Med. Virol..

[2] Jensen J.E., Becker G.L., Jackson J.B., Rysavy M.B. (2024). Human Papillomavirus and Associated Cancers: A Review. Viruses.

[3] World Health Organization Human Papillomavirus and Cancer. https://www.who.int/news-room/fact-sheets/detail/human-papilloma-virus-and-cancer.

[4] Alrefai E.A., Alhejaili R.T., Haddad S.A. (2024). Human Papillomavirus and Its Association With Cervical Cancer: A Review. Cureus.

[5] McBride A.A. (2024). Human Malignancies Associated with Persistent HPV Infection. Oncol..

[6] Wu J., Jin Q., Zhang Y., Ji Y., Li J., Liu X., Duan H., Feng Z., Liu Y., Zhang Y. (2025). Global Burden of Cervical Cancer: Current Estimates, Temporal Trend and Future Projections Based on the GLOBOCAN 2022. J. Natl. Cancer Cent..

[7] Fan X., He W., Zhang Q., Zhang B., Dong L., Li L., Liu X. (2024). Evaluation and Prediction Analysis of 3- and 5-Year Relative Survival Rates of Patients with Cervical Cancer: A Model-Based Period Analysis. Cancer Control.

[8] National Cancer Institute Cervical Cancer Prognosis and Survival Rates—NCI. https://www.cancer.gov/types/cervical/survival.

[9] American Cancer Society Cervical Cancer Survival Rates|Cancer 5 Year Survival Rates. https://www.cancer.org/cancer/types/cervical-cancer/detection-diagnosis-staging/survival.html.

[10] Manso L., Ramchandani-Vaswani A., Romero I., Sánchez-Lorenzo L., Bermejo-Pérez M.J., Estévez-García P., Fariña-Madrid L., García García Y., Gil-Martin M., Quindós M. (2024). SEOM-GEICO Clinical Guidelines on Cervical Cancer (2023). Clin. Transl. Oncol..

[11] World Health Organization Human Papillomavirus Vaccines: WHO Position Paper, December 2022. https://www.who.int/publications/i/item/who-wer9750-645-672.

[12] Basoya S., Anjankar A. (2022). Cervical Cancer: Early Detection and Prevention in Reproductive Age Group. Cureus.

[13] Pathak P., Pajai S., Kesharwani H. (2022). A Review on the Use of the HPV Vaccine in the Prevention of Cervical Cancer. Cureus.

[14] Singh D., Vignat J., Lorenzoni V., Eslahi M., Ginsburg O., Lauby-Secretan B., Arbyn M., Basu P., Bray F., Vaccarella S. (2023). Global Estimates of Incidence and Mortality of Cervical Cancer in 2020: A Baseline Analysis of the WHO Global Cervical Cancer Elimination Initiative. Lancet Glob. Health.

[15] Shattock A.J., Johnson H.C., Sim S.Y., Carter A., Lambach P., Hutubessy R.C.W. (2024). Contribution of Vaccination to Improved Survivl and Health: Modelling 50 Years of the Expanded Programme on Immunization. Lancet.

[16] Lai L.Y., Arshad F., Areia C., Alshammari T.M., Alghoul H., Casajust P. (2022). Current Approaches to Vaccine Safety Using Observational Data: A Rationale for the EUMAEUS (Evaluating Use of Methods for Adverse Events Under Surveillance-for Vaccines) Study Design. Front. Pharmacol..

[17] Soper D. (2006). Reducing the Health Burden of HPV Infection through Vaccination. Infect. Dis. Obstet. Gynecol..

[18] Mahajan I., Kadam A., McCann L., Ghose A., Wakeham K., Dhillon N.S., Stanway S., Boussios S., Banerjee S., Priyadarshini A. (2024). Early Adoption of Innovation in HPV Prevention Strategies: Closing the Gap in Cervical Cancer. ecancermedicalscience.

[19] Tobaiqy M., MacLure K. (2024). A Systematic Review of Human Papillomavirus Vaccination Challenges and Strategies to Enhance Uptake. Vaccines.

[20] Castro F.G., Barrera M., Holleran Steiker L.K. (2010). Issues and Challenges in the Design of Culturally Adapted Evidence-Based Interventions. Annu. Rev. Clin. Psychol..

[21] Barbieri V., Wiedermann C.J., Lombardo S., Piccoliori G., Gärtner T., Engl A. (2024). Vaccine Hesitancy and Public Mistrust During Pandemic Decline: Findings from 2021 and 2023 Cross-Sectional Surveys in Northern Italy. Vaccines.

[22] World Health Organization Immunization Coverage. https://www.who.int/news-room/fact-sheets/detail/immunization-coverage.

[23] World Health Organization Global Strategy to Accelerate the Elimination of Cervical Cancer as a Public Health Problem. https://www.who.int/publications-detail-redirect/9789240014107.

[24] Kane M.A., Serrano B., De Sanjosé S., Wittet S. (2012). Implementation of Human Papillomavirus Immunization in the Developing World. Vaccine.

[25] Alodhialah A.M., Almutairi A.A., Almutairi M. (2024). Assessing Barriers to Cancer Screening and Early Detection in Older Adults in Saudi Arabia: A Mixed-Methods Approach to Oncology Nursing Practice Implications. Curr. Oncol..

[26] McQuaid E.L., Landier W. (2018). Cultural Issues in Medication Adherence: Disparities and Directions. J. Gen. Intern. Med..

[27] Santos A.C.d., Silva N.N.T., da Silva I.D.C.G., Carneiro M., Coura-Vital W., Lima A.A. (2025). Effectiveness of HPV Vaccina-Tion in Reducing Infection among Young Brazilian Women. BMC Infect. Dis..

[28] Waheed D.-N., Burdier F.R., Eklund C., Baussano I., Mariz F.C., Téblick L. (2023). An Update on One-Dose HPV Vaccine Studies, Immunobridging and Humoral Immune Responses. A Meeting Report. Prev. Med. Rep..

[29] Moher D., Liberati A., Tetzlaff J., Altman D.G., Group P. (2009). Preferred Reporting Items for Systematic Reviews and Meta-Analyses: The PRISMA Statement. PLoS Med..

[30] Page M.J., McKenzie J.E., Bossuyt P.M., Boutron I., Hoffmann T.C., Mulrow C.D., Shamseer L., Tetzlaff J.M., Akl E.A., Brennan S.E. (2021). The PRISMA 2020 Statement: An Updated Guideline for Reporting Systematic Reviews. BMJ.

[31] Amir-Behghadami M., Janati A.P. (2020). Intervention, Comparison, Outcomes and Study (PICOS) Design as a Framework to Formulate Eligibility Criteria in Systematic Reviews. Emerg. Med. J..

[32] Mendeley. https://www.mendeley.com/search/.

[33] Critical Appraisal Skills Programme, CASP. https://casp-uk.net/casp-tools-checklists/.

[34] Campbell M., McKenzie J.E., Sowden A., Katikireddi S.V., Brennan S.E., Ellis S. (2020). Synthesis without Meta-Analysis (SWiM) in Systematic Reviews: Reporting Guideline. BMJ.

[35] Fokom Domgue J., Dille I., Kapambwe S., Yu R., Gnangnon F., Chinula L. (2024). HPV Vaccination in Africa in the COVID-19 Era: A Cross-Sectional Survey of Healthcare Providers’ Knowledge, Training, and Recommendation Practices. Front. Public Health.

[36] Zhang C., Greengold J., Tackett S., Lentz C., Bennett W., McGuire M. (2023). Evaluation of Online Educational Curriculum on HPV Vaccination Practices among Adult Primary Care Providers. BMC Med. Educ..

[37] Sullivan-Blum Z.C., Brophy M., Didde R., Nagireddy R., Swagerty H., Weir S. (2022). PrEP Patient Attitudes, Beliefs and perceived Barriers Surrounding HPV Vaccination: A Qualitative Study of Semistructured Interviews with PrEP Patients in Primary Care Clinics in Kansas and Missouri. BMJ Open.

[38] Hecht M.L., BeLue R., Ray A., Hopfer S., Miller-Day M., Mckee F. (2022). HPV Vaccine Intent among Adult Women Receiving Care at Community Health Centers. J. Cancer Educ..

[39] Brandt H.M., Vanderpool R.C., Curry S.J., Farris P., Daniel-Ulloa J., Seegmiller L. (2019). A Multi-Site Case Study of Community-Clinical Linkages for Promoting HPV Vaccination. Hum. Vaccines Immunother..

[40] Thompson E.L., Vamos C.A., Sappenfield W.M., Straub D.M., Daley E.M. (2016). Relationship Status Impacts Primary Reasons for Interest in the HPV Vaccine among Young Adult Women. Vaccine.

[41] Crann S.E., Barata P.C., Mitchell R., Mawhinney L., Thistle P., Chirenje Z.M., Stewart D.E. (2016). Healthcare Providers’ Perspec-Tives on the Acceptability and Uptake of HPV Vaccines in Zimbabwe. J. Psychosom. Obstet. Gynecol..

[42] Canfell K., Egger S., Velentzis L.S., Brown J.D., O’Connell D.L., Banks E., Sitas F. (2015). Factors Related to Vaccine Uptake by Young Adult Women in the Catch-up Phase of the National HPV Vaccination Program in Australia: Results from an Observa-Tional Study. Vaccine.

[43] Lei J., Ploner A., Elfström K.M., Wang J., Roth A., Fang F. (2020). HPV Vaccination and the Risk of Invasive Cervical Cancer. N. Engl. J. Med..

[44] Tran N.T., Phan T.N.T., Pham T.T., Le T.T., Le H.M., Nguyen D.T. (2023). Urban-Rural Disparities in Acceptance of Human Papillomavirus Vaccination among Women in Can Tho, Vietnam. Ann. Ig..

[45] Raviglione M.C.B., Tediosi F., Villa S., Casamitjana N., Plasència A. (2023). Global Health Essentials.

[46] Karanja-Chege C.M. (2022). HPV Vaccination in Kenya: The Challenges Faced and Strategies to Increase Uptake. Front. Public Health.

[47] Ewongwo A., Sahor A.F., Ngwa W., Nwachukwu C. (2024). A Guide to Global Access to HPV Vaccination to All Women in Low- and Middle-Income Countries; a Minireview of Innovation and Equity. Front. Oncol..

[48] Ver A.T., Notarte K.I., Velasco J.V., Buac K.M., Nazareno J., Lozañes J.A. (2021). A Systematic Review of the Barriers to Implementing Human Papillomavirus Vaccination Programs in Low- and Middle-Income Countries in the Asia-Pacific. Asia Pac. J. Clin. Oncol..

[49] Nogueira-Rodrigues A., Flores M.G., Macedo Neto A.O., Braga L.A.C., Vieira C.M., Sousa-Lima R.M. (2022). HPV Vaccination in Latin America: Coverage Status, Implementation Challenges and Strategies to Overcome It. Front. Oncol..

[50] MacDonald S.E., Kenzie L., Letendre A., Bill L., Shea-Budgell M., Henderson R. (2023). Barriers and Supports for Uptake of Human Papillomavirus Vaccination in Indigenous People Globally: A Systematic Review. PLOS Glob. Public Health.

[51] Islam J.Y., Gurbani A., Ramos S., Morgan K., Kim C.J., Richter K.L. (2021). Health Care Provider Perceptions of Facilitators and Barriers to Human Papillomavirus Vaccination Delivery in Five Countries. Sex. Transm. Dis..

[52] Amponsah-Dacosta E., Kagina B.M., Olivier J. (2020). Health Systems Constraints and Facilitators of Human Papillomavirus Immunization Programmes in Sub-Saharan Africa: A Systematic Review. Health Policy Plan..

[53] Kutz J.M., Rausche P., Gheit T., Puradiredja D.I., Fusco D. (2023). Barriers and Facilitators of HPV Vaccination in Sub-Saharan Africa: A Systematic Review. BMC Public Health.

[54] Waheed D.E., Bolio A., Guillaume D., Sidibe A., Morgan C., Karafillakis E. (2023). Planning, Implementation, and Sustaining High Coverage of Human Papillomavirus (HPV) Vaccination Programs: What Works in the Context of Low-Resource Countries? Front. Public Health.

[55] Hakimi S., Lami F., Allahqoli L., Alkatout I. (2023). Barriers to the HPV Vaccination Program in the Eastern Mediterranean Region: A Narrative Review. J. Turk. Ger. Gynecol. Assoc..

[56] Xu M.A., Choi J., Capasso A., DiClemente R.J. (2024). Improving HPV Vaccination Uptake Among Adolescents in Low Resource Settings: Sociocultural and Socioeconomic Barriers and Facilitators. Adolesc. Health Med. Ther..

[57] Bogdanova A., Andrawos C., Constantinou C. (2022). Cervical Cancer, Geographical Inequalities, Prevention and Barriers in Resource Depleted Countries. Oncol. Lett..

[58] Peterson C.E., Silva A., Holt H.K., Balanean A., Goben A.H., Dykens J.A. (2020). Barriers and Facilitators to HPV Vaccine Uptake among US Rural Populations: A Scoping Review. Cancer Causes Control.

[59] Branda F., Pavia G., Ciccozzi A., Quirino A., Marascio N., Gigliotti S. (2024). Human Papillomavirus (HPV) Vaccination: Progress, Challenges, and Future Directions in Global Immunization Strategies. Vaccines.

[60] Giannone G., Giuliano A.R., Bandini M., Marandino L., Raggi D., Earle W. (2022). HPV Vaccination and HPV-Related Malignancies: Impact, Strategies and Optimizations toward Global Immunization Coverage. Cancer Treat. Rev..

[61] Tsu V.D., LaMontagne D.S., Atuhebwe P., Bloem P.N., Ndiaye C. (2021). National Implementation of HPV Vaccination Programs in Low-Resource Countries: Lessons, Challenges, and Future Prospects. Prev. Med..

[62] Guillaume D., Waheed D.E., Schleiff M., Muralidharan K.K., Vorsters A., Limaye R.J. (2024). Global Perspectives of Determinants Influencing HPV Vaccine Introduction and Scale-up in Low- and Middle-Income Countries. PLoS ONE.

[63] Wang H., Jiang Y., Wang Q., Lai Y., Holloway A. (2023). The Status and Challenges of HPV Vaccine Programme in China: An Exploration of the Related Policy Obstacles. BMJ Glob. Health.

[64] Aggarwal S., Agarwal P., Gupta N. (2024). A Comprehensive Narrative Review of Challenges and Facilitators in the Implementation of Various HPV Vaccination Program Worldwide. Cancer Med..

[65] Essa-Hadad J., Gorelik Y., Vervoort J., Jansen D., Edelstein M. (2024). Understanding the Health System Barriers and Enablers to Childhood MMR and HPV Vaccination among Disadvantaged, Minority or Underserved Populations in Middle- and High-Income Countries: A Systematic Review. Eur. J. Public Health.

[66] Grabert B.K., Heisler-MacKinnon J., Liu A., Margolis M.A., Cox E.D., Gilkey M.B. (2021). Prioritizing and Implementing HPV Vaccination Quality Improvement Programs in Healthcare Systems: The Perspective of Quality Improvement Leaders. Hum. Vaccines Immunother..

[67] Guillaume D., Meyer D., Waheed D.E., Schlieff M., Muralidharan K., Chou V.B., Limaye R. (2023). Factors influencing the prioritization of vaccines by policymakers in low- and middle-income countries: A scoping review. Health Policy Plan..

[68] Marques M.D., Sheel M. (2023). Addressing the social inequities of vaccination: An imperative to close the gap. Lancet Glob. Health.

[69] Ekezie W., Awwad S., Krauchenberg A., Karara N., Dembiński Ł., Grossman Z., del Torso S., Dornbusch H.J., Neves A., Copley S. (2022). For The ImmuHubs Consortium. Access to Vaccination among Disadvantaged, Isolated and Difficult-to-Reach Communities in the WHO European Region: A Systematic Review. Vaccines.

[70] Prem K., Cernuschi T., Malvolti S., Brisson M., Jit M. (2024). Optimal Human Papillomavirus Vaccination Strategies in the Context of Vaccine Supply Constraints in 100 Countries. eClinicalMedicine.

[71] Oberlin A.M., Rahangdale L., Chinula L., Fuseini N.M., Chibwesha C.J. (2018). Making HPV Vaccination Available to Girls Everywhere. Int. J. Gynaecol. Obstet..

[72] Sguanci M., Mancin S., Gazzelloni A., Diamanti O., Ferrara G., Morales Palomares S., Parozzi M., Petrelli F., Cangelosi G. (2024). The Internet of Things in the Nutritional Management of Patients with Chronic Neurological Cognitive Impairment: A Scop-Ing Review. Healthcare.

[73] Terzoni S., Ferrara P., Parozzi M., Colombani F., Mora C., Cilluffo S., Jeannette V.G., Destrebecq A., Pinna B., Lusignani M. (2023). Nurses’ role in the management of persons with chronic urogenital pelvic pain syndromes: A scoping review. Neurourol. Urodyn..

[74] Gazineo D., Parozzi M., Ricco M., Savini S., Ferrara G., Anastasi G., Cangelosi G., Godino L., Andreoli D. (2025). Relational skills of nephrology and dialysis nurses in clinical care settings: A scoping review and stakeholder consultation. Nurse Educ. Pract..

[75] Mieronkoski R., Azimi I., Rahmani A.M., Aantaa R., Terävä V., Liljeberg P., Salanterä S. (2017). The Internet of Things for Basic Nursing Care—A Scoping Review. Int. J. Nurs. Stud..

[76] Scuri S., Tesauro M., Petrelli F., Argento N., Damasco G., Cangelosi G., Nguyen C.T.T., Savva D., Grappasonni I. (2022). Use of an Online Platform to Evaluate the Impact of Social Distancing Measures on Psycho-Physical Well-Being in the COVID-19 Era. Int. J. Environ. Res. Public Health.

[77] Lee G.A., Durante A., Baker E.E., Vellone E., Caggianelli G., Dellafiore F., Khan M., Khatib R. (2023). Patients’ perceptions on the facilitators and barriers using injectable therapies in dyslipidaemia: An empirical qualitative descriptive international study. J Adv. Nurs..

[78] Prado L., Allemann S., Viprey M., Schott A.M., Dediu D., Dima A.L. (2023). Toward an Interdisciplinary Approach to Constructing Care Delivery Pathways from Electronic Health Care Databases to Support Integrated Care in Chronic Conditions. Systematic Review of Quantification and Visualization Methods. J. Med. Internet Res..

[79] Cangelosi G., Mancin S., Morales Palomares S., Pantanetti P., Quinzi E., Debernardi G., Petrelli F. (2024). Impact of School Nurse on Managing Pediatric Type 1 Diabetes with Technological Devices Support: A Systematic Review. Diseases.

[80] Gawande M.S., Zade N., Kumar P., Gundewar S., Weerarathna I.N., Verma P. (2025). The Role of Artificial Intelligence in Pandemic Responses: From Epidemiological Modeling to Vaccine Development. Mol. Biomed..

