# Supplementary material for: Papillomavirus Vaccination Programs and Knowledge Gaps as Barriers to Implementation: A Systematic Review
Source: Vaccines (Basel). 2025 Apr 25;13(5):460. doi: 10.3390/vaccines13050460 (PMC12116001; doi:10.3390/vaccines13050460)
Supplement: Supplementary file 1 [file vaccines-13-00460-s001.zip › Supplementary File S2.pdf]

Supplementary File S2

(Search strategy update February 02, 2025)

Pubmed/Medline Strategy

(((((((((((("Communication Barriers"[Mesh]) OR "Social Isolation"[Mesh]) OR "Deaf Culture"[Mesh]) OR "Resource-Limited Settings"[Mesh]) OR "Social Segregation"[Mesh]) OR ("Socioeconomic Factors"[Mesh] OR "Economic Factors"[Mesh])) OR "Health Knowledge, Attitudes, Practice"[Mesh]) OR ("Health Risk Behaviors"[Mesh] OR "Health Status Indicators"[Mesh] OR "Risk Assessment"[Mesh] OR "Risk Factors"[Mesh])) OR ("Patient Acceptance of Health Care"[Mesh] OR "Patient Satisfaction"[Mesh])) OR "Developing Countries"[Mesh]) OR ("Healthcare Disparities"[Mesh])) AND (((((((((((("Mass Vaccination"[Mesh]) OR "Vaccination Coverage"[Mesh]) OR "Immunization Programs"[Mesh]) OR ("Public Health"[Mesh] OR "Nurses, Public Health"[Mesh] OR "Public Health Surveillance"[Mesh])) OR "Primary Health Care"[Mesh]) OR "Primary Prevention"[Mesh]) OR "Mass Screening"[Mesh]) OR "Tertiary Healthcare"[Mesh]) OR "Healthcare Disparities"[Mesh]) OR "Health Policy"[Mesh]) OR "Community Health Services"[Mesh]) OR "Universal Health Care"[Mesh])) AND (((ouverture vaccinale HPV) OR (hpv vaccine campaign)) OR ("Papillomavirus Vaccines"[Mesh])) Filters: in the last 10 years, Clinical Trial, Controlled Clinical Trial, Multicenter Study, Observational Study, Pragmatic Clinical Trial, Randomized Controlled Trial

Total results: 160

SCOPUS

TITLE-ABS-KEY ("communication barriers") OR TITLE-ABS-KEY ("social isolation") OR TITLE-ABS-KEY ("deaf culture") OR TITLE-ABS-KEY ("resource-limited settings") OR TITLE-ABS-KEY ("social segregation") OR TITLE-ABS-KEY ("socioeconomic factors") OR TITLE-ABS-KEY ("economic factors") OR TITLE-ABS-KEY ("health knowledge, attitudes, practice") OR TITLE-ABS-KEY ("health risk behaviors") OR TITLE-ABS-KEY ("health status indicators") OR TITLE-ABS-KEY ("risk assessment") OR TITLE-ABS-KEY ("risk factors") OR TITLE-ABS-KEY ("patient acceptance of healthcare") OR TITLE-ABS-KEY ("patient satisfaction") OR TITLE-ABS-KEY ("developing countries") OR TITLE-ABS-KEY ("healthcare disparities") AND TITLE-ABS-KEY ("mass vaccination") OR TITLE-ABS-KEY ("vaccination coverage") OR TITLE-ABS-KEY ("immunization programs") OR TITLE-ABS-KEY ("public health") OR TITLE-ABS-KEY ("nurses, public health") OR TITLE-ABS-KEY ("public health surveillance") OR TITLE-ABS-KEY ("primary health care") OR TITLE-ABS-KEY ("primary prevention") OR TITLE-ABS-KEY ("mass screening") OR TITLE-ABS-KEY ("tertiary healthcare") OR TITLE-ABS-KEY ("health policy") OR TITLE-ABS-KEY ("community health services") OR TITLE-ABS-KEY ("universal health care") AND TITLE-ABS-KEY ("ouverture vaccinale HPV") OR TITLE-ABS-KEY ("hpv vaccine campaign") OR TITLE-ABS-KEY ("papillomavirus vaccines") AND PUBYEAR > 2013 AND PUBYEAR < 2025 AND (LIMIT-TO (DOCTYPE , "ar"))

Total results: 859

EMBASE

('communication barriers'/exp OR 'social isolation'/exp OR 'deaf culture'/exp OR 'resource-limited settings'/exp OR 'social segregation'/exp OR 'socioeconomic factors'/exp OR 'economic factors'/exp OR 'health knowledge, attitudes, practice'/exp OR 'health risk behaviors'/exp OR 'health status indicators'/exp OR 'risk assessment'/exp OR 'risk factors'/exp OR 'patient acceptance of health care'/exp OR 'patient satisfaction'/exp OR 'developing countries'/exp OR 'healthcare disparities'/exp) AND ('mass vaccination'/exp OR 'vaccination coverage'/exp OR 'immunization programs'/exp OR 'public health'/exp OR 'nurses, public health'/exp OR 'public health surveillance'/exp OR 'primary health care'/exp OR 'primary prevention'/exp OR 'mass

screening'/exp OR 'tertiary healthcare'/exp OR 'healthcare disparities'/exp OR 'health policy'/exp OR 'community health services'/exp OR 'universal health care'/exp) AND ('ouverture vaccinale hpv' OR 'hpv vaccine campaign' OR 'papillomavirus vaccines'/exp) AND [2015-2025]/py AND ('case control study'/de OR 'clinical trial'/de OR 'clinical trial topic'/de OR 'cohort analysis'/de OR 'comparative study'/de OR 'controlled study'/de OR 'cross sectional study'/de OR 'intervention study'/de OR 'longitudinal study'/de OR 'major clinical study'/de OR 'multicenter study'/de OR 'observational study'/de OR 'pilot study'/de OR 'prospective study'/de OR 'quantitative study'/de OR 'randomized controlled trial'/de OR 'randomized controlled trial topic'/de OR 'retrospective study'/de)

Total results: 1.100
