# Supplementary material for: Papillomavirus Vaccination Programs and Knowledge Gaps as Barriers to Implementation: A Systematic Review
Source: Vaccines (Basel). 2025 Apr 25;13(5):460. doi: 10.3390/vaccines13050460 (PMC12116001; doi:10.3390/vaccines13050460)
Supplement: Supplementary file 1 [file vaccines-13-00460-s001.zip › Supplementary File S3.pdf]

Supplementary File S3: CASP Check List

Casp Checklist for RCT

**Reference: Hecht et al. / 2022**				
**Section A Is the basic study design valid for a randomised controlled trial?**				
**N**	**Item**	**Yes**	**Can't Tell**	**No**
**1**	Did the study address a clearly formulated research question?	**X**		
**2**	Was the assignment of participants to interventions randomised?	**X**		
**3**	Were all participants who entered the study accounted for at its conclusion?	**X**		
**Section B Was the study methodologically sound?**				
**N**	**Item**	**Yes**	**Can't Tell**	**No**
**4a**	Were the participants 'blind' to intervention they were given?		**X**	
**4b**	Were the investigators 'blind' to the intervention they were giving to participants?		**X**	
**4c**	Were the people assessing/analysing outcome/s 'blinded'?		**X**	
**5**	Were the study groups similar at the start of the randomised controlled trial?	**X**		
**6**	Apart from the experimental intervention, did each study group receive the same level of care (that is, were they treated equally)?	**X**		
**Section C: What are the results?**				
**N**	**Item**	**Yes**	**Can't Tell**	**No**
**7**	Were the effects of intervention reported comprehensively?	**X**		
**8**	Was the precision of the estimate of the intervention or treatment effect reported?	**X**		
**9**	Do the benefits of the experimental intervention outweigh the harms and costs?			**X**
**Section D: Will the results help locally?**				
**N**	**Item**	**Yes**	**Can't Tell**	**No**
**10**	Can the results be applied to your local population/in your context?	**X**		
**11**	Would the experimental intervention provide greater value to the people in your care than any of the existing interventions?	**X**		
**APPRAISAL SUMMARY**				
**Positive/Methodologically sound**	**Negative/Relatively poor methodology**	**Unknowns**		
**X**				

Casp Checklist for Qualitative Studies

**Reference: Sullivan-Blum et al. / 2022**				
**Section A: Are the result valid?**				
**N**	**Item**	**Yes**	**Can't Tell**	**No**
**1**	Was there a clear statement of the aims of the research?	**X**		
**2**	Is a qualitative methodology appropriate?	**X**		
**3**	Was the research design appropriate to address the aims of the research?	**X**		

**4**	Was the recruitment strategy appropriate to the aims of the research?	**X**		
**5**	Was the data collected in a way that addressed the research issue?	**X**		
**6**	Has the relationship between researcher and participants been adequately considered?	**X**		
**Section B: What are the results?**				
**N**	**Item**	**Yes**	**Can't Tell**	**No**
**7**	Have ethical issues been taken into consideration?	**X**		
**8**	Was the data analysis sufficiently rigorous?	**X**		
**9**	Is there a clear statement of findings?	**X**		
**Section C: Will the results help locally?**				
**N**	**Item**	**Yes**	**Can't Tell**	**No**
**10**	How valuable is the research?	**X**		
**APPRAISAL SUMMARY**				
**Positive/Methodologically sound**	**Negative/Relatively poor methodology**			**Unknowns**
**X**				

**Reference: Crann et al. / 2016**				
**Section A: Are the result valid?**				
**N**	**Item**	**Yes**	**Can't Tell**	**No**
**1**	Was there a clear statement of the aims of the research?	**X**		
**2**	Is a qualitative methodology appropriate?	**X**		
**3**	Was the research design appropriate to address the aims of the research?	**X**		
**4**	Was the recruitment strategy appropriate to the aims of the research?	**X**		
**5**	Was the data collected in a way that addressed the research issue?	**X**		
**6**	Has the relationship between researcher and participants been adequately considered?	**X**		
**Section B: What are the results?**				
**N**	**Item**	**Yes**	**Can't Tell**	**No**
**7**	Have ethical issues been taken into consideration?	**X**		
**8**	Was the data analysis sufficiently rigorous?	**X**		
**9**	Is there a clear statement of findings?	**X**		
**Section C: Will the results help locally?**				
**N**	**Item**	**Yes**	**Can't Tell**	**No**
**10**	How valuable is the research?	**X**		
**APPRAISAL SUMMARY**				
**Positive/Methodologically sound**	**Negative/Relatively poor methodology**			**Unknowns**
**X**				

**Reference: Brandt et al. / 2019**				
**Section A: Are the result valid?**				
**N**	**Item**	**Yes**	**Can't Tell**	**No**
**1**	Was there a clear statement of the aims of the research?	**X**		
**2**	Is a qualitative methodology appropriate?	**X**		
**3**	Was the research design appropriate to address the aims of the research?	**X**		
**4**	Was the recruitment strategy appropriate to the aims of the research?	**X**		
**5**	Was the data collected in a way that addressed the research issue?	**X**		
**6**	Has the relationship between researcher and participants been adequately considered?	**X**		
**Section B: What are the results?**				
**N**	**Item**	**Yes**	**Can't Tell**	**No**
**7**	Have ethical issues been taken into consideration?	**X**		
**8**	Was the data analysis sufficiently rigorous?	**X**		
**9**	Is there a clear statement of findings?	**X**		
**Section C: Will the results help locally?**				
**N**	**Item**	**Yes**	**Can't Tell**	**No**
**10**	How valuable is the research?	**X**		
**APPRAISAL SUMMARY**				
**Positive/Methodologically sound**	**Negative/Relatively poor methodology**			**Unknowns**
**X**				

Casp Checklist for Cohort Studies

**Reference: Thomson et al. / 2016**				
**Section A: Are the result valid?**				
**N**	**Item**	**Yes**	**Can't Tell**	**No**
**1**	Did the study address a clearly focused issue?	**X**		
**2**	Was the cohort recruited in an acceptable way?	**X**		
**3**	Was the exposure accurately measured to minimise bias?	**X**		
**4**	Was the outcome accurately measured to minimise bias?	**X**		
**5a**	Have the authors identified all important confounding factors?	**X**		
**5b**	Have they taken account of the confounding factors in the design and/or analysis?	**X**		
**6a**	Was the follow up of subjects complete enough?	**X**		
**6b**	Was the follow up of subjects long enough?	**X**		
**Section B: What are the results?**				
**N**	**Item**	**Yes**	**Can't Tell**	**No**
**7**	What are the results of this study?	**X**		
**8**	How precise are the results?	**X**		
**9**	Do you believe the results?	**X**		

Section C: Will the results help locally?				
**N**	**Item**	**Yes**	**Can't Tell**	**No**
**10**	Can the results be applied to the local population?	**X**		
**11**	Do the results of this study fit with other available evidence?	**X**		
**12**	What are the implications of this study for practice?	**X**		
**APPRAISAL SUMMARY**				
**Positive/Methodologically sound**	**Positive/Methodologically sound**			**Unknowns**
**X**				

Reference: Zhang et al. / 2023				
**Section A: Are the result valid?**				
**N**	**Item**	**Yes**	**Can't Tell**	**No**
**1**	Did the study address a clearly focused issue?	**X**		
**2**	Was the cohort recruited in an acceptable way?	**X**		
**3**	Was the exposure accurately measured to minimise bias?	**X**		
**4**	Was the outcome accurately measured to minimise bias?	**X**		
**5a**	Have the authors identified all important confounding factors?	**X**		
**5b**	Have they taken account of the confounding factors in the design and/or analysis?	**X**		
**6a**	Was the follow up of subjects complete enough?	**X**		
**6b**	Was the follow up of subjects long enough?	**X**		
**Section B: What are the results?**				
**N**	**Item**	**Yes**	**Can't Tell**	**No**
**7**	What are the results of this study?	**X**		
**8**	How precise are the results?	**X**		
**9**	Do you believe the results?	**X**		
**Section C: Will the results help locally?**				
**N**	**Item**	**Yes**	**Can't Tell**	**No**
**10**	Can the results be applied to the local population?	**X**		
**11**	Do the results of this study fit with other available evidence?	**X**		
**12**	What are the implications of this study for practice?	**X**		
**APPRAISAL SUMMARY**				
**Positive/Methodologically sound**	**Positive/Methodologically sound**			**Unknowns**
**X**				

Reference: Canfell et al. / 2015				
**Section A: Are the result valid?**				
**N**	**Item**	**Yes**	**Can't Tell**	**No**
**1**	Did the study address a clearly focused issue?	**X**		
**2**	Was the cohort recruited in an acceptable way?	**X**		
**3**	Was the exposure accurately measured to minimise bias?	**X**		

**4**	Was the outcome accurately measured to minimise bias?	**X**		
**5a**	Have the authors identified all important confounding factors?	**X**		
**5b**	Have they taken account of the confounding factors in the design and/or analysis?	**X**		
**6a**	Was the follow up of subjects complete enough?	**X**		
**6b**	Was the follow up of subjects long enough?	**X**		
**Section B: What are the results?**				
**N**	**Item**	**Yes**	**Can't Tell**	**No**
**7**	What are the results of this study?	**X**		
**8**	How precise are the results?	**X**		
**9**	Do you believe the results?	**X**		
**Section C: Will the results help locally?**				
**N**	**Item**	**Yes**	**Can't Tell**	**No**
**10**	Can the results be applied to the local population?	**X**		
**11**	Do the results of this study fit with other available evidence?	**X**		
**12**	What are the implications of this study for practice?	**X**		
**APPRAISAL SUMMARY**				
**Positive/Methodologically sound**	**Positive/Methodologically sound**			**Unknowns**
**X**				

Casp Checklist for Descriptive/Cross-Sectional Studies

**Reference: Domgue et al. / 2024**				
**Section A: Are the result valid?**				
**N**	**Item**	**Yes**	**Can't Tell**	**No**
**1**	Did the study address a clearly focused issue?	**X**		
**2**	Did the authors use an appropriate method to answer their question?	**X**		
**3**	Were the subjects recruited in an acceptable way?	**X**		
**4**	Were the measures accurately measured to reduce bias?	**X**		
**5**	Were the data collected in a way that addressed the research issue?	**X**		
**6**	Did the study have enough participants to minimise the play of chance?	**X**		
**7**	How are the results presented and what is the main result?	**X**		
**8**	Was the data analysis sufficiently rigorous?	**X**		
**9**	Is there a clear statement of findings?	**X**		
**10**	Can the results be applied to the local population?	**X**		
**11**	How valuable is the research?	**X**		
**APPRAISAL SUMMARY**				
**Positive/Methodologically sound**	**Positive/Methodologically sound**			**Unknowns**
**X**				
